# A Man with Labile Blood Pressure

**DOI:** 10.1371/journal.pmed.0040111

**Published:** 2007-04-24

**Authors:** Ronald C. W Ma, Kwok Hing Yiu, Edward H. C Wong, Kin Hung Liu, Joseph Y. S Chan, Chun Chung Chow, Clive S Cockram

**Affiliations:** 1 Department of Medicine and Therapeutics, Prince of Wales Hospital, Chinese University of Hong Kong, Shatin, New Territories, Hong Kong; 2 Department of Diagnostic Radiology and Organ Imaging, Prince of Wales Hospital, Chinese University of Hong Kong, Shatin, New Territories, Hong Kong

## Abstract

Ronald Ma and colleagues discuss the differential diagnosis and management of a patient who presented with recurrent episodes of chest discomfort, palpitations, and labile blood pressure.

## Presentation of Case

A 53-year-old man was admitted to hospital in September 2005 with chest pain. He had a history of nasopharyngeal carcinoma in 1986, for which he was treated with fractionated radiotherapy (62.5 Gy total). His blood pressure (BP) was 100/70 mmHg at the time of diagnosis. He was in remission following radical radiotherapy, but had partial hypopituitarism for which he required thyroxine replacement. Six months prior to admission, he complained of chest discomfort, was noted to be hypertensive in clinic with BP 156/93 mmHg and pulse rate of 107 bpm, and was started on atenolol. Renal function tests, electrolytes, fasting lipids, thyroid-stimulating hormone, and free T_4_ (thyroxine) were normal. Baseline electrocardiogram (ECG) was unremarkable. An exercise tolerance test was negative. On the day of admission, the patient described retrosternal chest tightness lasting 10 minutes. This was associated with palpitations and nausea. His medications were felodipine, propranolol, alprazolam, and thyroxine. On admission, he was markedly hypertensive (BP 182/123 mmHg). On examination, the patient was afebrile. His BP was persistently elevated. His pulse rate was 80 bpm. Cardiovascular examination was otherwise unremarkable. He had no audible carotid or abdominal bruits. Neurological examination was unremarkable. Fundoscopy was normal. Bedside urine analysis was normal. Plasma electrolytes, renal function, liver function, and amylase were all normal. Fasting glucose was 6.3 mmol/l. Heart size was normal on chest X-ray. ECG showed sinus rhythm (90 bpm) and incomplete right bundle branch block. The patient was initially treated for suspected acute coronary syndrome and was started on an intravenous nitrate infusion. Shortly afterwards, his BP abruptly dropped to 97/62 mmHg and the nitrate infusion was discontinued. Repeat ECG with right-sided chest leads did not show any evidence of right ventricular infarct. Serial troponin T tests were negative. On further questioning, he described a 6-month history of episodic chest discomfort, palpitations, and sweating. He also described marked fluctuation in blood pressure with home readings around 80/40 mmHg and markedly elevated readings in clinic. During admission, frequent fluctuation in BP with a systolic BP (SBP) between 106–226 mmHg and a diastolic BP (DBP) between 62–114 mmHg was noted. This was accompanied by a resting tachycardia with frequent fluctuation of heart rate.

**Figure 1:**
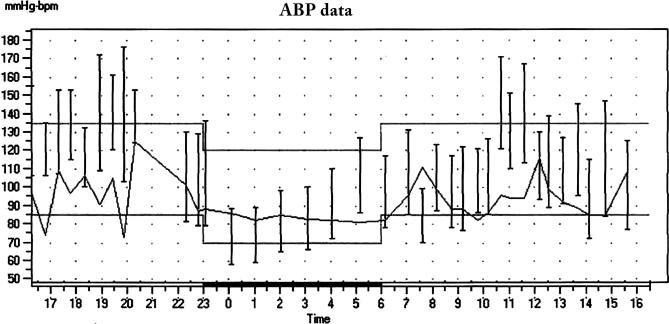
Ambulatory Blood Pressure Chart 24-hour ambulatory BP and pulse showing marked variability in BP, with frequent surges of BP during daytime activities and lack of heart rate correlation.

**Figure 2:**
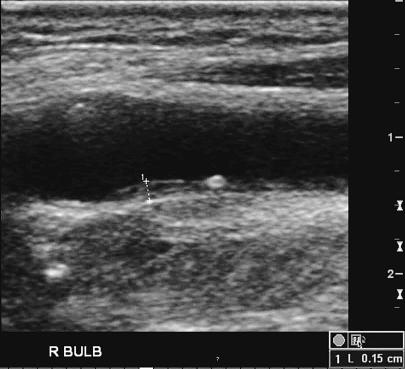
Duplex Ultrasound of the Right Carotid Artery The image shows diffuse thickening of the intimal wall (intimal medial thickness of 0.15 cm at right bulb, 0.16 cm at left bulb) with focal plaques present at bilateral distal common carotid arteries at bulb regions.

Phaeochromocytoma was suspected on the basis of the patient's presenting symptoms and labile blood pressure, but repeated collections for urinary catecholamines were normal during admission despite documented paroxysms during the collections. The patient denied use of any recreational drugs. Psychiatric assessment did not support a diagnosis of anxiety disorder or panic attacks. Twenty-four hour ambulatory BP monitoring revealed a mean asleep BP of 107/69 mmHg with mean heart rate 85 bpm but frequent surges of BP during waking daily activities, with mean daytime BP 139/94 mmHg and average awake heart rate of 95 bpm (Figure 1). In view of the widely fluctuant blood pressure and resting tachycardia, autonomic failure was also considered. The patient had absent heart rate response to standing, with reduced heart rate response to the Valsalva manoeuvre (RR ratio 1.07, age-matched lower limit of normal: 1.24), consistent with parasympathetic failure. There was no postural hypotension, and mental arithmetic with the serial-7 test caused a significant rise in BP, suggesting there was no sympathetic failure. A bolus intravenous injection of 50 μg nitroprusside induced a significant drop in blood pressure, but did not cause any heart rate changes. Likewise, a rise in blood pressure induced with a bolus injection of 50 μg phenylephrine was not accompanied by a change in heart rate, supporting a diagnosis of baroreflex failure. Review of his medical history confirmed extensive irradiation to both sides of the neck during treatment of his nasopharyngeal carcinoma, and this was considered the likely cause of his baroreflex failure. In support of this theory, a carotid duplex ultrasound showed diffuse thickening of the intimal wall with focal plaques affecting the bulb regions of both carotid arteries (Figure 2).

Clonidine was prescribed to the patient, which led to improvement in his symptoms as well as his BP. A repeat 24-hour ambulatory BP monitor 4 weeks later showed a mean BP of 126/83 mmHg with only one surge of BP to 175/100 mmHg and only 30% of SBP readings and 33% of DBP >140/90 mmHg. The patient remained well in July 2006 on clonidine 0.1 mg TDS.

## Discussion

The presence of paroxysmal hypertension should always alert health-care providers to the possibility of a catecholamine-secreting phaeochromocytoma. The diagnosis is supported by the demonstration of elevated plasma or urine catecholamines or metanephrines [1]. However, if this diagnosis is not confirmed, an alternative explanation needs to be sought. Other causes of paroxysmal hypertension include labile hypertension, hyperthyroidism, renovascular hypertension, seizure disorder, migraine, alcohol withdrawal, drugs such as cocaine, amphetamines, or clozapine [2], carcinoid syndrome, panic disorder, and baroreflex failure [3]. In some cases of paroxysmal hypertension, no obvious cause could be identified, though it has been suggested that careful psychosocial interviewing may uncover repressed emotional distress that patients may not be aware of [4].

**Figure 3:**
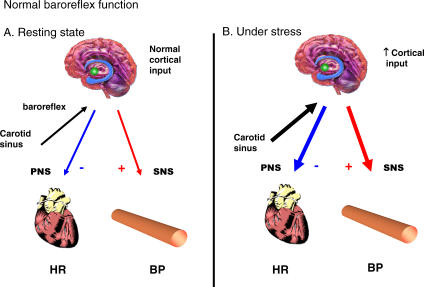
Normal Baroreflex Function With normal baroreflex function, there is appropriate balance of sympathetic and parasympathetic tone to maintain BP and heart rate, under both normal resting conditions (A), and during arousal, when there is increased cortical input (B). BP, blood pressure; HR, heart rate; PNS, parasympathetic nervous system; SNS, sympathetic nervous system.

**Figure 4:**
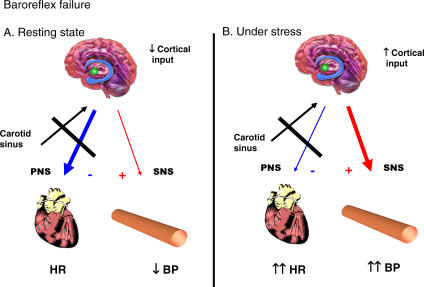
Pathophysiology of Baroreflex Failure In baroreflex failure, there is interrupted afferent baroreflex input, as well as efferent parasympathetic output. This results in decreased BP with no change in heart rate in the resting state, when there is reduced cortical input (A). However, increased sympathetic activity and lack of efferent parasympathetic activity during arousal will lead to marked elevation of BP and HR (B). BP, blood pressure; HR, heart rate; PNS, parasympathetic nervous system; SNS, sympathetic nervous system.

Arterial baroreceptors provide a tonic inhibitory influence on sympathetic tone (Figure 3), failure of which can result in excessive fluctuation in blood pressure. Baroreflex failure can result from abnormalities in vascular baroreceptors, the glossopharyngeal or vagal nerves, or the brainstem. This results in interrupted afferent baroreflex input from carotid sinus to nucleus tractus solitarii with accompanied interrupted efferent parasympathetic output to the heart and vessels (Figure 4). Affected patients may exhibit marked and acute variations in blood pressure [5]. Causes include carotid surgery, brainstem stroke, afferent sensory neuropathy, or neck trauma [6]. It is also a recognized late complication of neck irradiation [7], which may accelerate the development of carotid arteriosclerosis, causing splinting of the carotid mechanoreceptors in rigidified arterial walls and thereby disrupting baroreflex regulation of cardiovagal and sympathetic outflow [7].

A diagnosis of baroreflex failure should be suspected in patients with excessive excursion of heart rate during normal daily activities, often with marked fluctuation in blood pressure. The diagnosis is supported by the finding of absent bradycardia in response to a pressor such as phenylephrine or absent tachycardia in response to a depressor such as nitroprusside. Baroreflex failure can often be distinguished from pure autonomic failure. The presentation of autonomic failure is typically dominated by orthostatic hypotension, and often associated with supine hypertension and postprandial hypotension. Labile hypertension and episodic tachycardia are not usually observed. The presentation of baroreflex failure, on the other hand, often resembles that of phaeochromocytoma, with labile hypertension and episodic tachycardia, whilst orthostatic hypotension is rarely seen [6]. Subjects with baroreflex failure often have a low blood pressure at rest. In rare cases where there is selective baroreflex failure with interrupted afferent baroreflex input from the carotid sinus to the nucleus tractus solitarii with intact efferent sympathetic and parasympathetic output to the heart and great vessels, the condition can also lead to malignant vagotonia [8].

Treatment is aimed at reducing the frequency and magnitude of the life-threatening surges in blood pressure. In addition, a secondary goal of therapy is to attenuate symptomatic hypotensive episodes. Behavioural therapy with relaxation training can help by reducing sympathetic surges. Clonidine, a centrally and peripherally acting α-adrenoceptor agonist, can dampen sympathetic activation to limit the extent of pressor surges. Treatment with clonidine reduces the frequency of attacks and attenuates the blood pressure and heart rate changes during an attack [9]. In patients whose condition has been well controlled for prolonged periods, clonidine may be tapered off and replaced by high doses of benzodiazepines. In rare cases with predominant hypotension, low doses of fludrocortisone may be required [6].

## Supporting Information

Table S1 Summary of results of evaluation (37 KB DOC)

Text S2 Data from autonomic function tests (77 KB PPT)

**Learning Points**

- Presence of paroxysmal hypertension may indicate an underlying condition causing secondary hypertension.
- Labile hypertension may be due to hyperthyroidism, renovascular hypertension, seizure disorder, migraine, alcohol withdrawal, carcinoid syndrome, panic disorder, baroreflex failure, or drugs such as cocaine, amphetamines, or clozapine.
- Baroreflex failure should be considered in the differential diagnosis of labile hypertension, especially in subjects with a history of neck injury or neck irradiation.
- Treatment of baroreflex failure is aimed at reducing blood pressure surges and avoiding hypotensive episodes.

## Abbreviations

BP: blood pressure
bpm: beats per minute
DBP: diastolic blood pressure
ECG: electrocardiogram
Gy: gray
SBP: systolic blood pressure

## References

[pmed-0040111-b001] Lenders JW, Eisenhofer G, Mannelli M, Pacak K (2005). Phaeochromocytoma. Lancet.

[pmed-0040111-b002] Li JK, Yeung VT, Leung CM, Chow CC, Ko GT (1997). Clozapine: A mimicry of phaeochromocytoma. Aust N Z J Psychiatry.

[pmed-0040111-b003] Timmers HJ, Wieling W, Karemaker JM, Lenders JW (2004). Baroreflex failure: A neglected type of secondary hypertension. Neth J Med.

[pmed-0040111-b004] Mann SJ (1999). Severe paroxysmal hypertension (pseudopheochromocytoma): understanding the cause and treatment. Arch Intern Med.

[pmed-0040111-b005] Smit AA, Timmers HJ, Wieling W, Wagenaar M, Marres HA (2002). Long-term effects of carotid sinus denervation on arterial blood pressure in humans. Circulation.

[pmed-0040111-b006] Ketch T, Biaggioni I, Robertson R, Robertson D (2002). Four faces of baroreflex failure: Hypertensive crisis, volatile hypertension, orthostatic tachycardia, and malignant vagotonia. Circulation.

[pmed-0040111-b007] Sharabi Y, Dendi R, Holmes C, Goldstein DS (2003). Baroreflex failure as a late sequela of neck irradiation. Hypertension.

[pmed-0040111-b008] Jordan J, Shannon JR, Black BK, Costa F, Ertl AC (1997). Malignant vagotonia due to selective baroreflex failure. Hypertension.

[pmed-0040111-b009] Robertson D, Hollister AS, Biaggioni I, Netterville JL, Mosqueda-Garcia R (1993). The diagnosis and treatment of baroreflex failure. N Engl J Med.

