# Supplementary material for: A Man with Labile Blood Pressure
Source: PLoS Med. 2007 Apr 24;4(4):e111. doi: 10.1371/journal.pmed.0040111 (PMC1855694; doi:10.1371/journal.pmed.0040111)
Supplement: Table S1 — (37 KB DOC) [file pmed.0040111.st001.doc]

Table 1. Summary of results of evaluation

| Investigation | Results | Interpretation |
| --- | --- | --- |
| 1. Drug history |  | Drug causes unlikely. Denies use of recreational drugs |
| 2. Thyroid function tests |  | Hyperthyroidism excluded |
| 3. Urinary catecholamines | Two 24-hour urine collections performed during paroxysms show normal urinary catecholamines | Phaeochromocytoma unlikely |
| 4. Computed tomography of brain | Normal | No intracranial lesion |
| 5. Psychiatry assessment |  | Panic attack or anxiety disorder unlikely |
| 6. Twenty-four hour ambulatory BP | Mean asleep BP of 107/69mmHg, mean heart rate 85 bpm. Frequent surges of BP during waking daily activities, with mean daytime BP 139/94 and average awake heart rate of 95 bpm | Frequent BP surges during waking activities. Resting tachycardia raising possibility of autonomic dysfunction. Ambulatory BP pattern also consistent with baroreflex failure |
| 7. Autonomic function testing |  |  |
| -Valsalva manoeuvre | Reduced heart rate response.  RR ratio 1.07 (age-matched lower limit of normal: 1.24) | Impaired cardiovagal function |
| -Mental arithmetic with serial 7s | Significant rise in BP within 60 seconds | Preserved sympathetic efferent pathways |
| -Postural change | No postural hypotension | Preserved sympathetic adrenergic function |
| -Phenylephrine (50µg bolus) | Resulted in adequate rise in BP by >20mmHg, but did not lead to a reflex decrease in HR | Baroreflex failure with parasympathetic failure but preserved sympathetic function |
| -Nitroprusside (50µg bolus) | Decrease in BP by >20mmHg but no significant increase in HR observed | Baroreflex failure with parasympathetic failure |
| -Propranolol  (1mg/min IV infusion for 10mins) | Significant decrease in HR seen | Preserved sympathetic regulation of heart rate |
| -Atropine (0.04mg/kg bolus) | Minimal increase in HR | Impaired parasympathetic control of heart rate |
| 8. Carotid duplex ultra-sound | Bilateral diffuse thickening of intimal wall with focal plaques | Premature atherosclerosis due to neck irradiation |
