# Supplementary material for: A Man with Labile Blood Pressure
Source: PLoS Med. 2007 Apr 24;4(4):e111. doi: 10.1371/journal.pmed.0040111 (PMC1855694; doi:10.1371/journal.pmed.0040111)
Supplement: Text S2 — (77 KB PPT) [file pmed.0040111.sd001.ppt]

## Slide 1
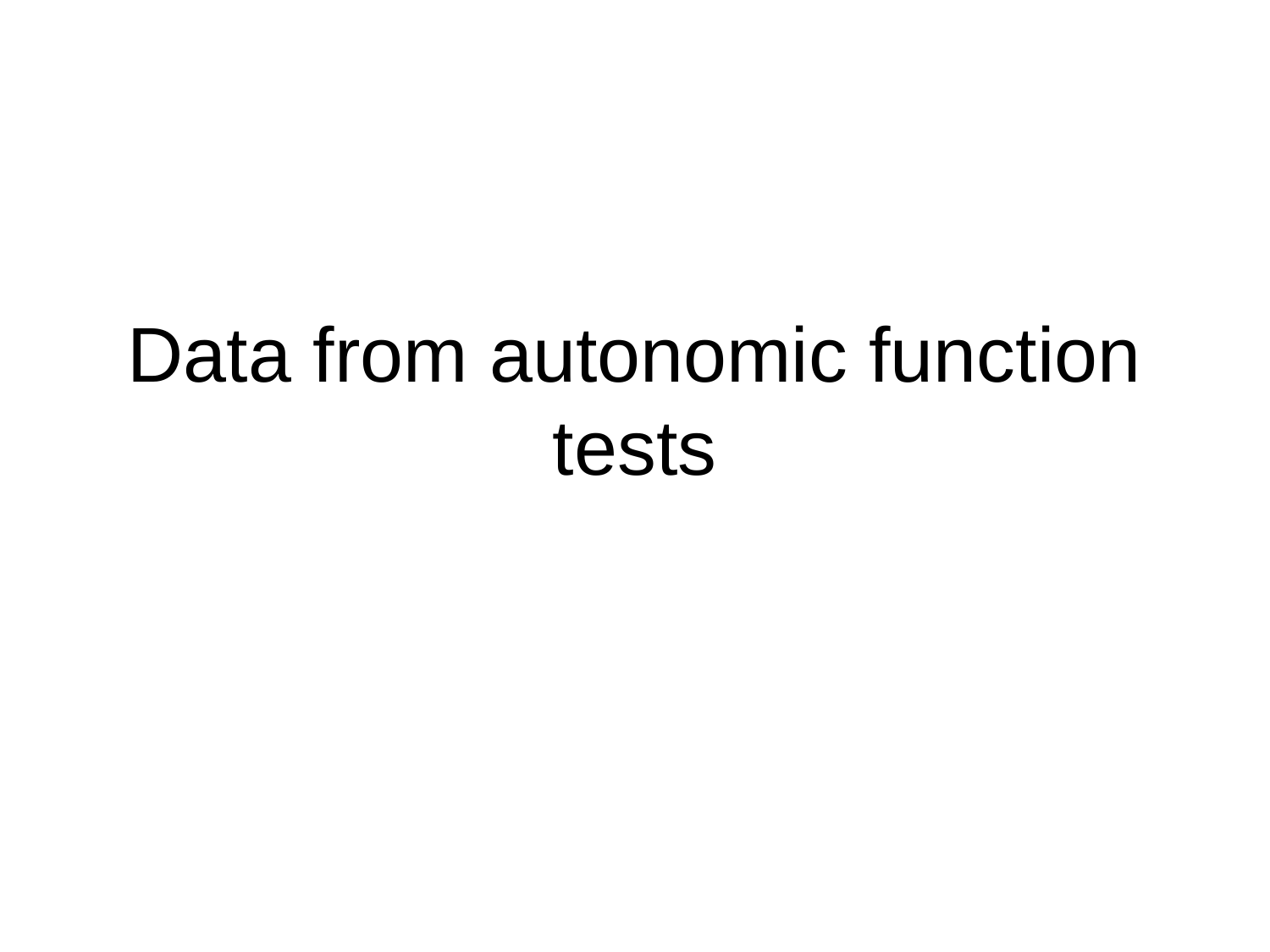

# Data from autonomic function tests

## Slide 2
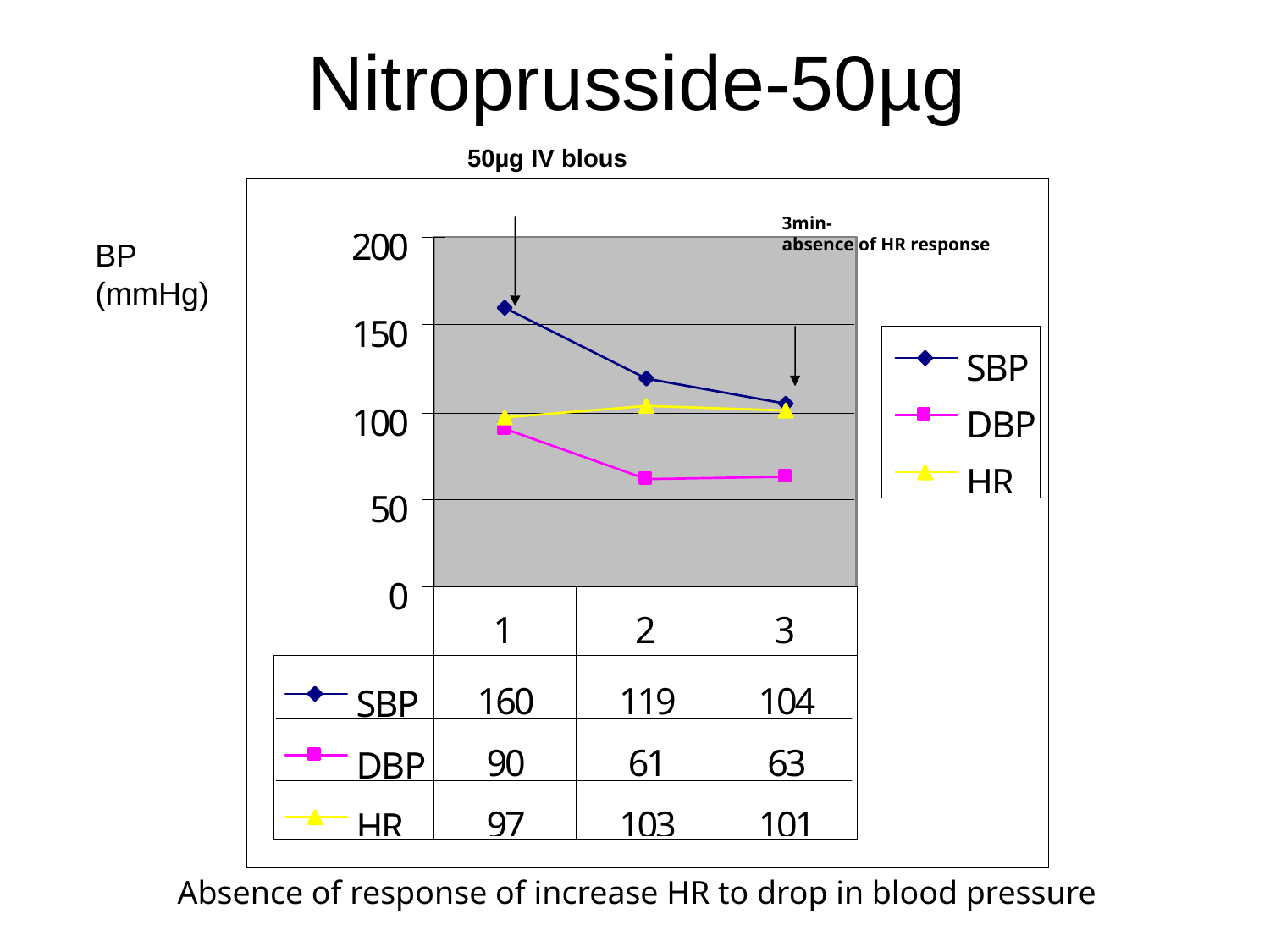

# Nitroprusside-50µg
50µg IV blous
3min-
absence of HR response
BP
(mmHg)
Absence of response of increase HR to drop in blood pressure

## Slide 3
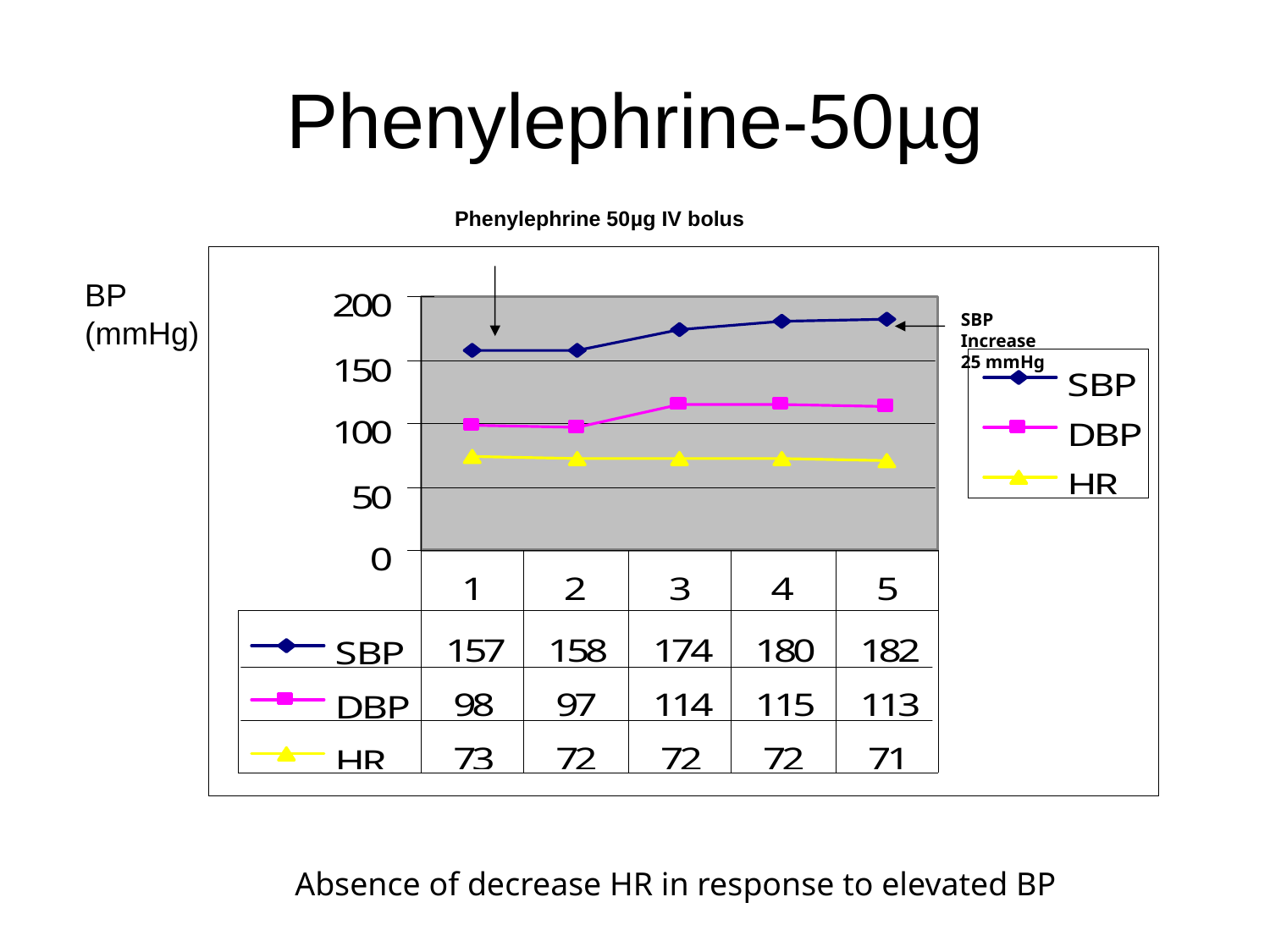

# Phenylephrine-50µg
Phenylephrine 50µg IV bolus
BP
(mmHg)
SBP
Increase
25 mmHg
Absence of decrease HR in response to elevated BP

## Slide 4
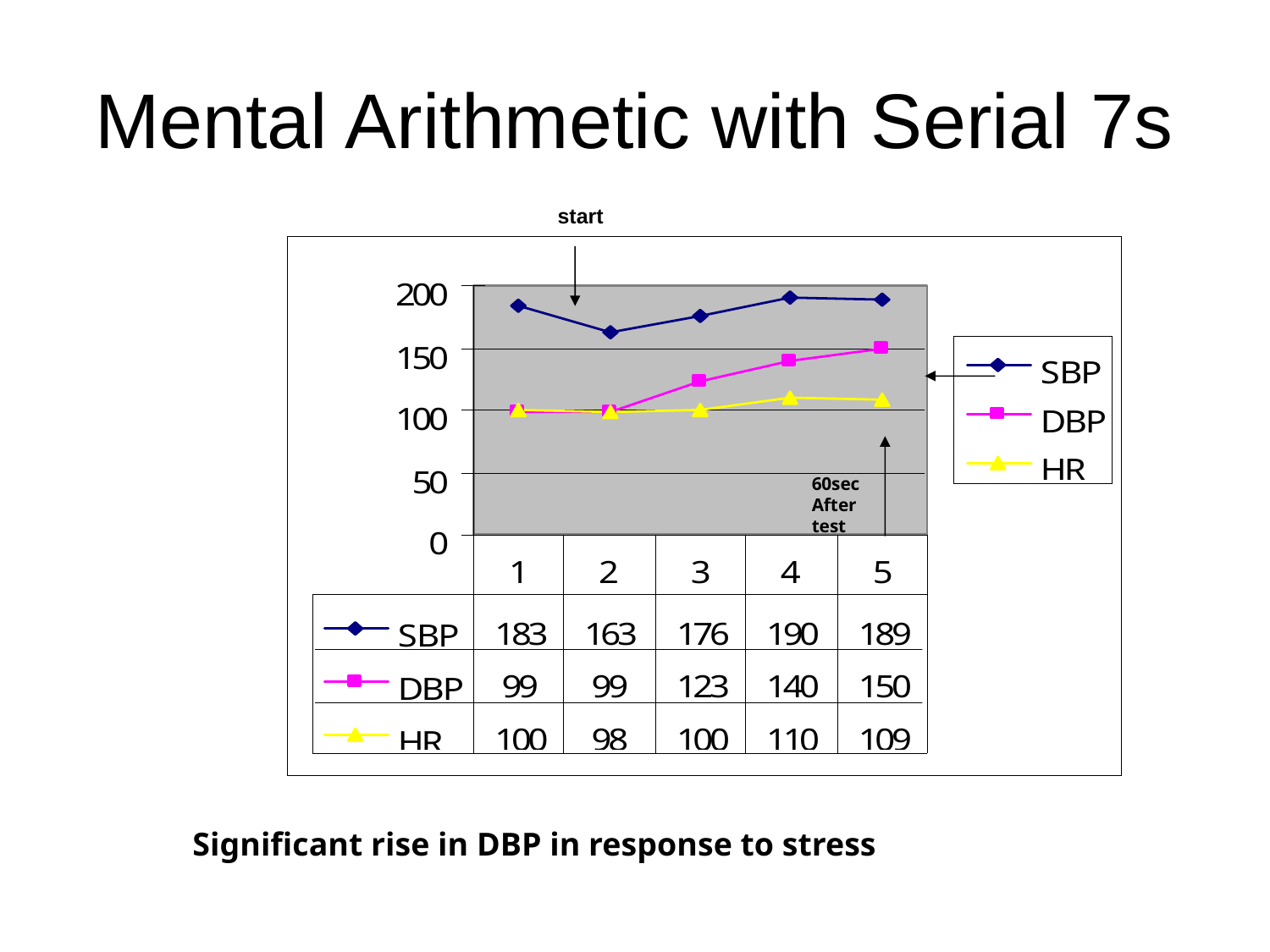

# Mental Arithmetic with Serial 7s
start
60sec
After
test
Significant rise in DBP in response to stress

## Slide 5
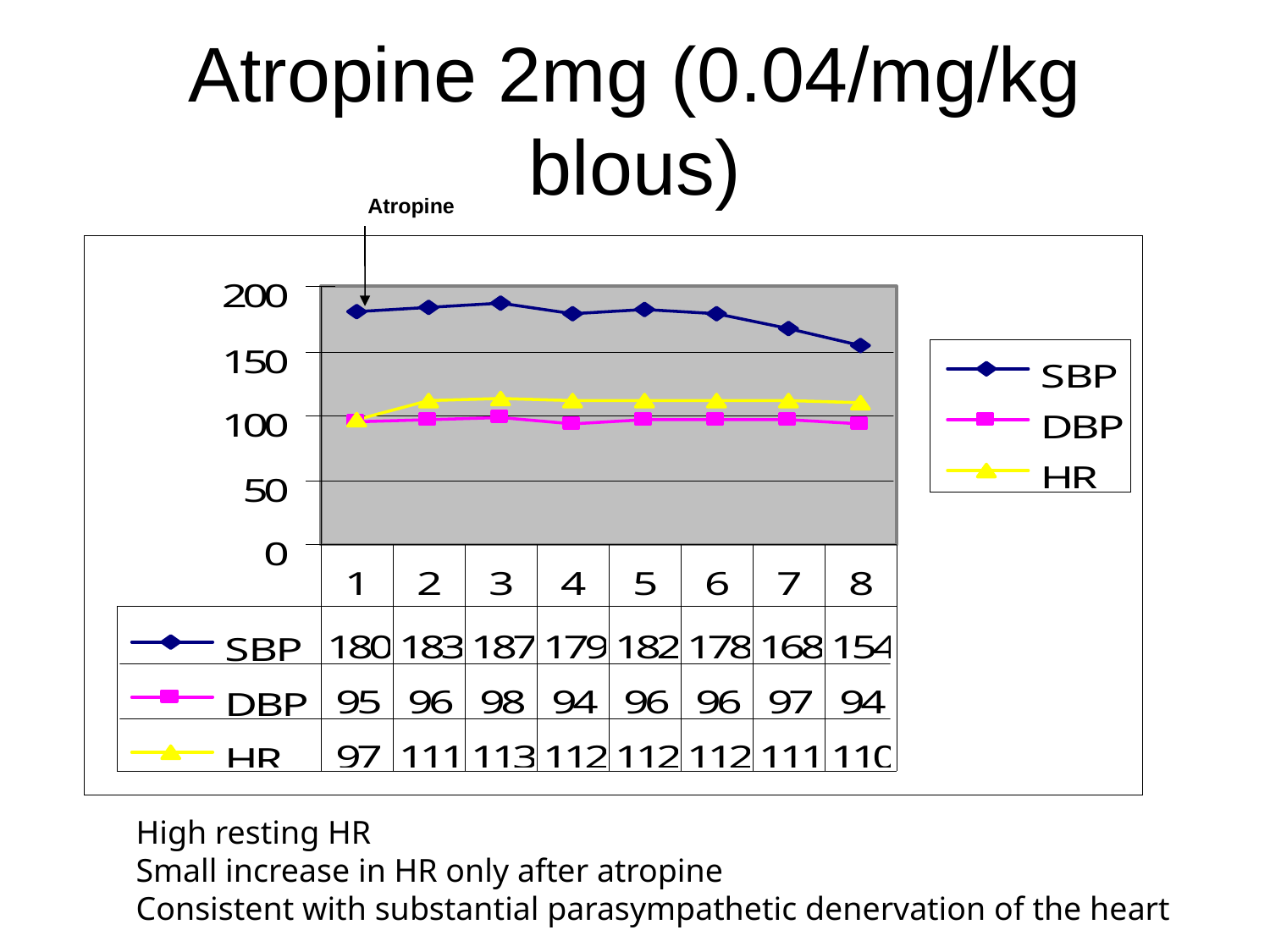

# Atropine 2mg (0.04/mg/kg blous)
Atropine
High resting HR
Small increase in HR only after atropine
Consistent with substantial parasympathetic denervation of the heart

## Slide 6
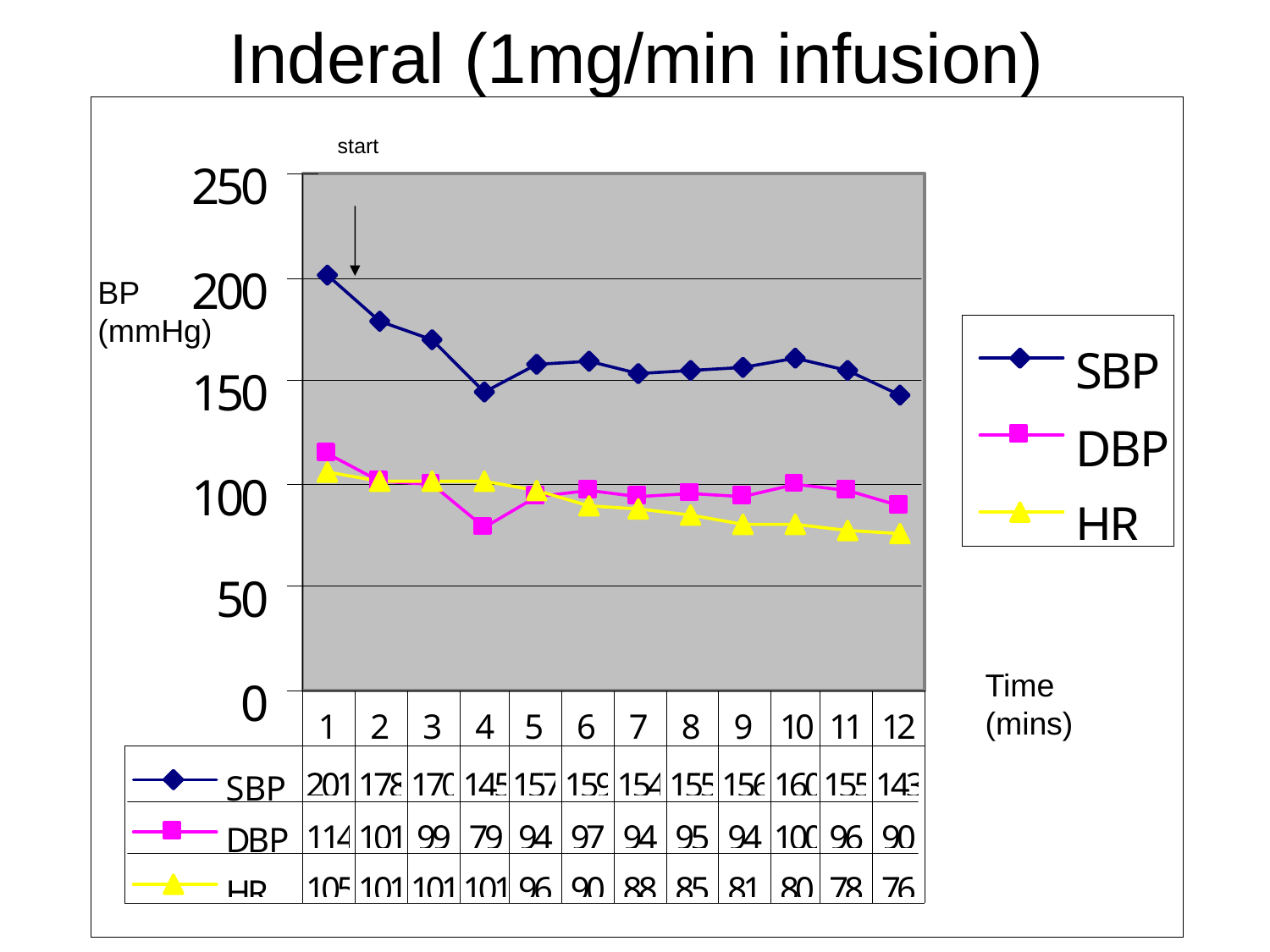

# Inderal (1mg/min infusion)
start
BP
(mmHg)
Time
(mins)
